# Clinical significance of microRNA-200 and let-7 families expression assessment in patients with ovarian cancer

**DOI:** 10.3332/ecancer.2021.1249

**Published:** 2021-06-14

**Authors:** Severyn Ferneza, Markiyan Fetsych, Roman Shuliak, Halyna Makukh, Natalia Volodko, Roman Yarema, Taras Fetsych

**Affiliations:** 1 Department of Oncology and Radiology FPGE, Danylo Halytsky Lviv National Medical University, Hasheka 2A str., Lviv 79000, Ukraine; 2 Department of Microinvasive Surgery, Lviv State Regional Oncology Treatment and Diagnostic Center, Hasheka 2A str., Lviv 79000, Ukraine; 3 Institute of Hereditary Pathology, National Academy of Medical Sciences of Ukraine, Lviv 79000, Ukraine

**Keywords:** microRNA, ovarian cancer, biomarkers

## Abstract

Ovarian cancer (OC) represents the most lethal malignancy in gynaecologic oncology practice and shows a high recurrence rate due to its early chemoresistance to first-line chemotherapy. Yet, timely selection of the correct treatment strategy is likely to prolong a patient’s survival. MicroRNAs (miRNAs) are a class of short non-coding RNAs responsible for the expression of 30%–60% of human genes. In numerous studies, miRNAs have been used to provide the overall prognosis for patients and analyse the process’s prevalence and responses to chemotherapy. In particular, miRNAs as markers for predicting the sensitivity of OC to platinum- and taxane-based chemotherapeutics can significantly improve the treatment efficacy. This article highlights two families of miRNAs: miR-200 and let-7, which are promising for further research on OC and its chemosensitivity.

## Introduction

Today, ovarian cancer (OC) ranks among all cancers as the 7th leading cause of morbidity and mortality: 6.6 per 100,000 women are diagnosed with OC; 3.9 per 100,000 women die from OC [1, 2]. OC is the 5th leading cause of death in gynaecologic oncology patients [1].

Early-stage OC is highly curable, which is supported by the high 5-year survival rate of such patients [3]. While the 5-year survival rate makes up 29%, OC is diagnosed in the late stages in most cases. Only 15% of cases are diagnosed in the first stage when the 5-year survival rate reaches 92% [2]. The overall 5-year survival does not exceed 45%–47% [2] and is characterised by the high recurrence rate – up to 20%–25% on the stages I–II, and up to 70% when the disease is discovered in the late stages of its development. About 25% of all relapses occur within 6 months after the treatment completion; such ovarian tumours are considered chemoresistant and have an unfavourable prognosis [3]. Therefore, quick and reliable cancer prognostic approaches are essential at this stage.

The major scientific achievement in the OC study of the past decades was the definition of OC as a heterogeneous group of diseases within one morphological variant. By this definition, all epithelial tumours fall into two groups based on the course and prognosis of the disease, namely: type I – with more favourable prognosis (low-grade serous and endometrioid carcinomas, clear cell and mucinous OC, Brenner’s tumour), and type II with less favourable prognosis (high-grade serous OC, undifferentiated carcinoma and mesodermal tumours) [4]. This discovery was based on molecular genetics and later was implemented in clinical practice. In 2014, WHO and International Federation of Gynecology and Obstetrics (FIGO) revised its classification and updated it by classifying the serous OC as high and low malignant [5]. Hence, each of them was characterised by different genetic mutations, response to chemotherapy and differential prognosis [4]. An advanced analysis of the genome and transcriptome in OC samples, within the Cancer Genome Atlas project, allowed to outline four transcriptional subtypes of high-grade OC (mesenchymal, immunoreactive, differentiated and proliferative), which differ by the duration of patient's survival and the response to treatment three subtypes of microRNAs (miRNAs) [6]. In this respect, the results of the molecular genetic research linking miRNA and OC progression can be crucial for the development, implementation and efficiency assessment of the new treatment methods and the selection of patients for their application [7].

Discovered recently, miRNAs are known as a class of short non-coding RNAs (22 nucleotides). miRNAs can be found in tumour tissue and body fluids and regulate the expression of around 30% of human genes [8]. miRNAs are able to regulate the tumour’s response to treatment and, accordingly, affect the prognosis for a patient. According to recent studies, the expression level of some miRNAs affects the tumour resistance to chemotherapy [8] and radiation therapy [9]. Although a significant volume of data supports the miRNA role as an indicator for cancer prognosis during treatment [8], more information and methodology are needed to describe this phenomenon fully. For example, miRNAs within one family often show different properties in the process of oncogenesis, and their expression in blood and tumour tissue requires more detailed study. miRNAs of two families: miR-200 and let-7 are most frequently discussed in the publications on OC topic. Thus, the purpose of this review is to summarise and analyse the results of these studies and evaluate the possibility of clinical application of the specific miRNA expression.

## Role of microRNA-200 and let-7 members in clinical outcome

### miRNA correlates with cancer progression

#### microRNA-200 family

Out of all miRNAs known up to now, miRNA-200 family has been proven to play an essential role in carcinogenesis regulation. However, there are fundamental differences in how each member of this group functions. The family members in the miRNA of group 200 are typically located on two loci of different chromosomes, namely miR-200a, miR-200b, miR-429 are located together on chromosome 1, whereas miR-200c and miR-141 – on chromosome 12 [10]. Hu *et al* [11] suggest that part of miRNA-200 family, which are located on chromosome 1, has the possibility of co-expression and regulation. It is likely that miRs located on chromosome 1 have a similar impact on OC carcinogenesis. It may also appear that miRNA-200 family members located on chromosome 12 might have a similar tendency to co-regulation and co-expression.

miRNA-200 family is responsible for managing epithelial-mesenchymal transition (EMT) and its reverse process – mesenchymal-epithelial transition (MET), which can lose their normal functioning and lead to carcinogenesis under influence of dysregulated miRs. Zinc finger E-box-binding homeobox 1 (ZEB1) and Zinc finger E-box-binding homeobox 2 (ZEB2) genes are directly responsible for regulation of EMT and MET processes via downregulation of E-cadherin – the facilitator of intercellular binding and cell migration [12]. miR-200 family and ZEB1/ZEB2 control each other via mutual negative feedback – a regulatory mechanism by which miRNA-200 level can downregulate the two genes and vice versa [13]. The disruption of this process can lead to the failure in cell plasticity and develop into cancer metastasis. Interestingly, under some circumstances, miR-200c can downregulate cell proliferation, migration and invasion despite low E-cadherin levels [14]. In experiments on mice, a high level of miR-200c expression at the initial stages of tumour formation led to a significantly better tumour regression than after restoring the miR-200c levels in already formed tumour [15]. The expression of miR-200c was also observed in the inflammatory component of the cell population, which proves its involvement in the regulation of inflammation and, consequently, invasion [12]. Depending on the location of the HuR RNA-binding protein in the miR-200c cell, miR-200c indirectly alters its function by stimulating or inhibiting Tubulin beta chain 3 gene (TUBB3) expression [16]. Therefore, in some patients with a high level of miR-200c and TUBB3, the prognosis was worse: both recurrence-free period and overall survival were shorter. Such mechanism of miRNA interaction with cellular proteins may explain the differences in the function of the same miRNAs concluded by different studies [16].

This statement was partially supported in several studies with tumour tissues and cell lines. However, there is no consensus on the role of miR-200a in the progression of OC. Several studies revealed an increased level of miR-200a tissue expression at the advanced stages (III–IV) of OC [17–19]. Instead, some other studies have shown that miR-200a expression decreases with the disease progression [20]. Suo *et al* [21] observed a significant increase of miRNA expression in patients with OC but did not provide any data showing the change in expression depending on the stage of the disease. Interestingly, miR-200a expression is present not only in OC-affected tissues. Its level is higher in comparison to tissues taken from healthy individuals [18] and benign ovarian tumours [13]. Similar observations were made about miR-200a expression in lymphatic tissues. Specifically, decreased level of miR-200a was observed in intact lymph nodes and increased – in metastatic lymph nodes [18, 21], indicating the miR-200a potential as a carcinogenic miRNA. In the same time, in several studies, higher miR-200a expression was characteristic of patients without lymph node metastases [13, 20] and increased – in patients with ascites occurrence [20]. These discrepancies are most likely caused by the differences in preservation technique used in each study: frozen tissue samples in some [13] and formalin-fixed paraffin-embedded (FFPE) OC tissue preservation in other studies [20]. One group does not provide information of how tissue samples were stored during the study. There is a high chance of miRNA degradation in formalin samples. Therefore, choosing the appropriate sample preservation method may be crucial for further analysis [22].

Among studied publications, very little data was found on the correlation between the miR-200c tumour tissue levels and the stage of OC progression. For instance, one group has stated that the expression of miR-200c is higher at stages I–II than stages III–IV and has a lower incidence of lymph node involvement [23]. On the contrary, another group has observed an increased level of miR-200c at the stages III–IV [19].

The expression of miR-200b in OC cells is higher as compared to healthy cells [24]. Different results were obtained from patients’ tumour tissue – it was noticed that miR-200b is overexpressed in the tumour tissue of patients with stage I OC and has low expression in stage III [25]. This proves miR-200b to be oncosuppressive miR, and its drop leads to cancer progression. Such differences in statements are most likely caused by different research material used in both studies.

Unlike the healthy cells, those affected by OC showed higher expression of miR-141 and miR-429 [26]. This statement was proved by the experiments on tumour tissue samples [11].

The level of miRNA-200 family may be a prognostic factor for patient survival. In particular, an increased tissue expression of miR-200a correlated with the lower overall survival and relapse-free period [19]. Simultaneous overexpression of miR-200a, c, b was observed in women with lower overall survival [27]. In the same time, hyperexpression of miR-200a appears to be typical in the case of overall and recurrence-free survival [25, 27]. Complementary, the patients with low tissue expression of miR-200a had lower overall survival [11, 20] and shorter recurrence-free survival [11], supporting the above studies (Table 1). Besides these results, other groups have reported reduced [13, 17] or increased [28, 29] tissue expression of miR-200a but did not investigate the correlation between the miRNA levels and patient survival. Finally, some investigations found no relationship between the miR-200a expression level and patient survival [28, 30]. Overall, these findings suggest that miR-200a expression patterns in most cases have been indicative of a carcinogenic process and may prove to have a clinical value for patient’s prognosis (Table 2). Yet, more research on this topic is required.

In many studies, high miR-200c expression in tumour tissue or cell lines has been observed [28, 31]. Other authors, however, indicate a decrease in miRNA expression [32, 33]. Also, the results regarding the dependence of the miR-200c expression level on the prognosis of patients are contradictory: some sources show that miR-200c expression is beneficial for patient survival (Table 3) [28, 31], while the others state the opposite (Table 4) [19, 31] One research group determined miRNA expression of miR-200c-3p only in women with high-grade serous and clear cell histological subtype of OC. They found a negative inverse correlation between miR-200c-3p level and prognosis for patients only in the serous subtype group [34]. These findings show the potential involvement of the miR-200c in cancer progression but require more detailed evidence to follow.

High miR-200b levels in serum [35] and tissue [19, 31] are associated with lower overall and relapse-free survival. These findings strictly indicate miR-200b as carcinogenic miR (Table 5).

Overexpression of miR-141 and miR-429 may also serve as a marker for the prediction of reduced recurrence-free and overall survival [31]. Another study supported this statement, in which higher expression of miR-429 in OC cells compared to healthy cells [24]. At the same time, some research indicates that miR-141 and miR-200a can be a positive prognostic factor for patients (Table 5) [27, 28]. Both groups of authors used different techniques to store collected material – this may again serve as a reason for results discrepancies.

No correlation was found between the tumour grade and miR-200 family expression in tumour tissue, except for miR-200b [19]. No relationship was found neither of miR-200a [20, 21] nor of miR-200c, b [19] between miR expression and tumour size. Overexpression of miR-200a was typical for women with high-grade serous OC [17, 18]. At the same time, another group pointed at the reduced expression of miR-200a in patients with high-grade OC as compared to those with low-grade [13]. Such discrepancies may occur due to the different tissue preservation technique. The amount of high- and low-grade groups also remains unclear. The relationship between the histological subtype of OC and miRNA expression also remains controversial. Most studies, however, indicate the absence of such correlation [13, 20]. In contrast, another research [36] have found an increased expression of miR-200a, c in serous, endometrioid and clear cell subtypes. These discrepancies are most likely caused by different tissue preservation technologies: FFPE was used [20], in another – snap frozen tissues [36]. One author does not provide their methodology. miR-200b is hyperexpressed in clear cell OC [34] as well as in its endometrioid and serous subtype [36]. Increased expression of miR-141 is typical for endometrioid and serous subtype of OC [16].

Overall, miR-200 family has diverse roles in carcinogenesis. Sample preservation techniques are the potential primary cause of data discrepancies in miR studies and should be consistent for reliable comparisons.

#### Let-7 family

Let-7 family was one of the first miRs discovered to have a suppressive impact on carcinogenesis [37]. The let-7 miRNA family consists of 12 members located at eight loci of seven chromosomes: chromosome 3 – let-7g; 9 – a-1, d, f-1; 11 – a-2; 12 – i; 19 – e; 21 – c; 22 – a-3, b; x – f-2, miR-98 [38]. As compared to the miRNA 200 family, the let-7 family is less studied, but its role in carcinogenesis remains undoubted. Dysregulation of the let-7 family has been found in medulloblastoma, breast cancer, OC, melanoma and non-small cell lung cancer, clearly showing the role of the miRNA family in the carcinogenesis of many malignancies [38]. Decreased expression of let-7e, let-7f, let-7d, let-7c, let-7a-e, let-7i, let-7a, let-7b in tumour samples and decreased let-7f, let-7d, let-7a-e in cell lines [39], decreased cell growth and reduced lymph node involvement [38] proves an oncosuppressive role of this miRNAs family (Table 6).

All studies we have looked into agree on a cancer-suppressive role of let-7i. As it turns out, its low expression in tissue is characteristic of patients with poor prognosis [40], short recurrence-free period [37]. Low let-7i expression in patients with OC [39] might indirectly indicate its oncosuppressive role as well. Despite this, the decreased let-7i expression has been associated with the depth of metastasis invasion into lymph nodes in patients with gastric cancer. It is possible that let-7i may play a similar role in patients with OC [41]. No data could be found to describe the correlation between let-7i expression and disease progression as well as the degree of tumour differentiation in patients with OC. However, being able to negatively regulate the expression of Toll-like receptor 4 (TLR4) and MyD88 genes, let-7i prolongs the periods of overall and progression-free survival [41].

Most researchers observe an increased let-7a expression in ovarian tumour tissue [42, 43]. In contrast, another research group detected low let-7a expression both *in vitro* and in tumour masses [39]. Both research groups used significantly varying sample sizes in their studies. Furthermore, the first research group did not use cell lines in their research. A number of studies showed that let-7a expression in clinic should be assessed in combination with Lin28B and Insulin-like growth factor 2 (IGF-II), as their simultaneous high expression resulted in more frequent recurrences and higher overall mortality in patients receiving platinum and paclitaxel treatment [44]. This, in turn, may testify about the negative regulation of OC cells sensitivity to drugs of the first-line chemotherapy. High let-7a-3 activity indirectly reduced overall survival by 40%, but this did not affect the progression of OC [44]. High Lin28B expression is a more accurate indicator of poor prognosis than the dysregulation of any other axis component. Low Lin28B expression correlated with the less aggressive OC, lower tumour malignancy and better chemotherapy response, whereas high IGF-II expression correlated with very low survival and sensitivity to paclitaxel, yet let-7a itself did not affect survival [43].

### miRNAs modulate chemotherapy response via gene regulation

#### miRNA-200 family

MiRs may affect tumour drug sensitivity through gene regulation. In particular, it has been determined that overexpression of miR-200a promotes sensitivity to paclitaxel by inhibiting the Mitogen-activated protein kinase 14 (MAPK14) gene and p38α protein [27]. An increased expression of miR-200a in cell lines was associated with taxane sensitivity. Interestingly, that growth in paclitaxel concentration has not significantly changed cellular chemosensitivity [29].

Whereas miR-200a possesses strictly chemosensitising abilities (Table 1), for miR-200c some discrepancies were found. Nevertheless, the level of miR-200c expression undoubtedly impacts the tumour response to platinum and taxanes. *In vitro* experiments showed that the expression of miR-200c drops 4–5-fold from its normal levels in tissues with observed resistance to paclitaxel. At the same time, miR-200c reduces the sensitivity of cells to carboplatin by increasing the sensitivity to taxanes [45]. This mechanism remains unclear. These results are consistent with those of Van Jaarsveld *et al* [46], who claim that hyperexpression of miR-200c causes little resistance to cisplatin. In contrast, Liu *et al* [33] indicate that decreased miR-200c expression correlates with decreased sensitivity to cisplatin *in vitro*, *in vivo*, and retrospectively, based on tissue samples from patients with OC. miR-200c inhibits TUBB3, thereby increasing the sensitivity of cells to cisplatin [16] and paclitaxel [14, 16]. This statement was supported in the study using tumour tissue samples – lower miR-200c expression was indicated in patients with a poorer response to first-line OC chemotherapy such as taxanes and platinum-based chemotherapy [28]. Similar mechanism of chemosensitivity regulation was found by another study, where miR-200c increased the efficacy of *in vitro* drugs, in particular, to paclitaxel (82%–85%), vincristine (33%–35%) and epothilone B (43%–50%), which all affect the microtubule system [47]. Thus, miR-200c evidently has a positive impact on tumour chemosensitivity to taxanes (Table 3). The estimated negative effect to platinum-based drugs could have been caused by the differences in cell lines used in the study by Brozovic *et al* [45]. It also appears that such discrepancy of miR-200c function might be caused by different position of HuR in cell, also mentioned by Prislei *et al* [16].

No direct impact on tumour sensitivity to taxanes by miR-200b was found. However, miR-200b has the same impact on class III β-tubulin as any other member of miR-200 family. Thus, it may have an indirect influence on OC sensitivity to taxanes [28]. Another study has determined that increased expression of miR-200b results in better sensitivity to cisplatin (Table 5) [33].

It has been discovered that hyperexpression of miR-141 promotes sensitivity to paclitaxel by inhibiting the MAPK14 gene and the p38α protein [27]. miR-141’s positive effect on OC sensitivity to taxanes was also supported by *in vitro* research [45]. Alike the miR-200c, miR-141 also reduces cellular sensitivity to carboplatin by increasing sensitivity to taxanes [45]. Decreased expression of miR-141 *in vitro* led to increased resistance to paclitaxel by 4–5 times, and vice versa – after the administration of miR-141, the sensitivity to paclitaxel [45] has increased. miR-141’s negative effect on cell-to-platinum sensitivity was supported by another group, pointing out that overexpression of miR-141 caused the resistance to platinum-based drugs by inhibiting the expression of Kelch-like ECH-associated protein 1. It appears that a negative feedback loop between cisplatin and miR-141 exists. Cisplatin caused a transient increase in miR-141 expression, the level of which returned to normal in 24 hours [46]. Same as for miR-200c, miR-141 undoubtedly has a positive impact on a chemosensitivity to taxanes (Table 5). The estimated negative effect to platinum-based drugs might be caused by different OC cell lines used in the study.

miR-429 indicates a favourable prognosis for patient treatment – it has been revealed that hyperexpression of miR-429 *in vitro* leads to increased cell sensitivity to cisplatin (Table 5) [48].

#### Let-7 family

Some sources define let-7i as a regulator of OC sensitivity to chemotherapeutics. In particular, low levels of let-7i expression in tissue and *in vitro* cause low sensitivity to cisplatin [37]. It was also observed that progesterone increases the expression of let-7i in SKOV-3 cell lines (ovarian cancer cell lines) and therefore is involved in cell proliferation and chemosensitivity, as concluded by the authors. Introducing let-7i in OVCAR-3 cell lines reduced cell viability by 36% and increased apoptosis by 34% [49]. let-7i included in a chimeric construct with the Mucin 1 aptamer increased the permeability of this miRNA into the cell and led to an increase in the efficiency (sensitivity) of paclitaxel [49]. Radiation therapy reduces the therapeutic effect of let-7 family miRNA observed during chemical treatment [50]. This relation has been discovered for lung cancer and may be consistent in OC as well.

The role of let-7a in OC chemosensitivity was determined in a study conducted by Lu *et al* [42]. The authors point out that hyperexpression of this miRNA can potentiate the effect of platinum drugs in a mono-regime, but can negatively affect the prognosis upon combination treatment (e.g. first-line carboplatin + paclitaxel). Some studies conclude that let-7a reduces the tumour sensitivity to paclitaxel. This was supported by another research, where low Lin28B expression (from let-7a – Lin28B – IGF-II axis) correlated with the less aggressive OC, lower tumour malignancy and better chemotherapy response. In contrast, high IGF-II expression correlated with low survival and sensitivity to paclitaxel (yet let-7a alone did not affect survival) [43]. Another group observed that let-7a decreases cellular sensitivity to paclitaxel, doxorubicin and interferon alpha by inhibiting caspase-3 and, consequently, decreased cell apoptosis [51]. This study was carried out on breast cancer cells and hepatocellular carcinoma cells. These findings, however, may be relevant in OC as well.

Among the let-7 family members, five are associated with cell culture chemoresistance. All of them are expressed differently depending on cell lines, in particular: let-7e – were increased in paclitaxel-resistant lines but had reduced expression in others. This may signify a negative regulation of let-7e cell sensitivity to taxanes [52]. The role of let-7e in chemosensitivity to cisplatin is confirmed by Xiao *et al* [53]. The researchers showed reduced let-7e expression in platinum-resistant cell lines and overexpression in platinum-sensitive cells [53]. Let-7e increases the concentration of cisplatin in the cell by 20.2% and inhibits tumour growth *in vivo* [54]. Let-7e increases cellular sensitivity to cisplatin by inhibiting the Enhancer of zeste homolog 2 gene responsible for Dicer expression, which, in turn, increases sensitivity to cisplatin [54]. The let-7 family also affects the sensitivity to taxanes upon the presence of Mitochondrial inner membrane protease subunit 1 gene – a direct target for let-7 in the cells. High levels of let-7g increase the sensitivity to taxanes and vinblastine, but do not affect the sensitivity to carboplatin. let-7g effects chemosensitivity through the indirect inhibition of Multidrug resistance mutation 1 (MDR1). Patients with high MDR1 expression had a shorter recurrence-free period as compared to those whose MDR1 expression was unchanged or reduced [55]. When MDR is absent, the sensitivity to chemotherapy seems to be independent of let-7g, proving their cumulative effect. Tumour expression of let-7d was lower during re-operation [55], apparently, due to a decrease in tumour mass. Decreased expression of let-7 family members was downregulated in OC, medulloblastoma and breast cancer [38].

Let-7 family members’ expression level is a prominent example of how the miRNAs tissue expression could be used as a predictor of chemotherapeutic response in OC (Table 6). A body of evidence suggests that this effect is mediated by this miRNA close regulation of gene expression. More data is required to fully describe the known and establish other connections between let-7 expression and treatment success.

### Extracellular role of miRNA in the assessment of cancer progression and chemosensitivity

#### miR-200 family

The expression level of miR-200a in serum has been a focus of many studies. All authors reported an increase of miRNA expression in the serum of OC patients independent of the stage [35, 56]. No correlation was established between the miR-200a expression in serum and the duration of overall or recurrence-free survival [30], tumour size [30, 57], serum Cancer antigen 125 (CA-125) [30, 57], the use of neoadjuvant treatment [30], tumour grade [56, 57] or the involvement of lymph nodes in the cancer process [35, 57]. However, there was a correlation between an increased miR-200a expression and the presence of distant metastases and the mucinous subtype of OC [57]. Interestingly, the expression of miR-200a in serum was higher in healthy women in comparision to the group of women with benign ovarian tumours [35].

Expression of miR-200c is also observed in the serum of patients with OC compared to healthy individuals [30]. Moreover, healthy women can be distinguished from those with OC by the level of serum miR-200c (accuracy – 0.79, and for CA-125 – 50%) [26, 56]. The level of this miRNA itself serves as a factor of the overall survival prediction: high miR-200c levels are observed in patients with longer overall survival [26]. Another research group provides opposite results – high expression level of miR-200c in serum is related to the lower overall survival and to a shorter relapse-free period [35]. No correlation between miRNA levels and patient age, tumour size, neoadjuvant treatment effect, overall survival or recurrence-free survival was found at all [30]. The former group used the whole serum samples in their study, whereas the latter extracted miRs’ exosomes, this could explain the differences in their results. miRNAs are represented in peripheral blood binded with proteins or in exosomes [58]. Thus, measurement of only exosome fraction may not represent the whole extracellular miRNA scope. Ethnical groups also differ by miRNA expression [59]. The number of samples in the one of studies was significantly smaller, which can also affect the overall conclusion.

The level of serum miR-200c correlates with the disease progression. Its expression decreases from stage I to stage IV of OC [26, 47]. Instead, Meng *et al* [56] and Zuberi *et al* [57] pointed at a gradual increase in miRNA expression along with the OC stage progression. Another study found no statistically significant increase in miR-200c expression in stages I–II but instead reported advanced stage miRNA overexpression [35]. Serum expression of miR-200c was higher in the case of the formation of secondary foci in the lymph nodes [35, 56], although Wang *et al* [60] did not observe such a relationship. The level of serum miR-200c was also going up as the remote formation of secondary foci progressed [57, 60]. The results provided by Gao *et al* [26] oppositely show that the level of serum miRNA was lower in the metastatic than in local OC. Among the above studies, only one reports the correlation between the miR-200c serum level and the CA-125 level [35]. Other researchers did not find such correlation for miRNA expression neither in the serum [30, 60] nor in the tissue [34]. The expression of miR-200c in serum varies depending on the histological subtype of epithelial OC (serous, mucinous, endometrioid, clear cell and undifferentiated) [26]. All these findings show potential role of miR-200c as a target of ‘liquid biopsy’. The reason for discrepancies found in studies most likely hides in different material approach – For instance (f.i.), Wang *et al* isolated exosomes from the serum, whereas Meng *et al* and Zuberi *et al* [57] used the whole serum. As well as nonhomogenous age groups used in studies might have had an impact on miRNA expression.

The expression of miR-200b is also observed in blood serum. Serum expression of miR-200b is sensitive to the ovarian tissue changes: its level is higher in women with benign ovarian tumours compared to healthy ovarian tissue and lower compared to tumour tissues in patients with OC [56]. Opposite results were presented by another study – miR-200b expression was higher in patients with OC [30]. The abundance of miR-200b expression is independent of the stage of the OC progression [56]. Another study shows the potential carcinogenic role of miR-200b by describing its upregulation at stages III–IV of OC and in women with affected lymph nodes [35]. These results were confirmed by Zuberi *et al* [57]. This study, however, failed to detect the significant increase of miR-200b expression in the serum of OC patients as a function of the disease progression; also, no correlation was found between the miR-200b expression and the level of CA-125, age, tumour grade, tumour size, menopausal status, haemoglobin concentration or absence of pregnancy [57]. Another study denies the correlation between the serum miR-200b levels and tumour grade [56], whereas its correlation with CA-125 levels is established [35]. Again, discrepancies in these reports were most likely caused by the measurements of miR-200b taken in exosomes versus the whole serum.

Both miR-141 and -429 in serum act as carcinogenic miRNAs. Zheng *et al* [61] have noticed that miR-141 is hypoexpressed in plasma. Another study reported that the serum miR-141 level: i) increases as a function of cancer progression; ii) is higher during metastatic spread and iii) correlates with the lower overall survival [26]. Unlike healthy women’s serum miR-429 levels, those of OC patients increase simultaneously with the disease progression and stage of disease. The serum level of this miRNA is statistically significantly correlated with the level of CA-125 in blood serum. Low expression of miR-429 has been shown to have a positive effect on overall survival, moreover increasing cell migration and invasion [62]. In contrast to this, Meng *et al* [35] did not detect miR-429 or -141 exosomes in blood serum. The authors measured miRNAs in exosomes, whereas other research groups used the whole serum for it.

Therefore, miR-200 family members, extracellular abundance may be indicative of an optimistic prognosis for the patient (Table 7). Most evidence has been reconciled from blood serum samples, which proves this object reliable for this analysis. Nevertheless, more studies concerning miRNA level in whole serum and exosomes should be done.

#### Let-7 family

Data on let-7 family expression in serum of the patients with OC is lower compared to the miR-200 family. However, all available results list let-7 blood expression as a positive prognostic factor for patients with OC. Let-7i expression was detected in serum, but the data on this topic is insufficient. In their study, Langhe *et al* [41] have found that let-7i in the blood is hypoexpressed in patients with OC.

The expression of let-7a in blood plasma was also detected but not fully described. Zheng *et al* [61] indicated a decreased miRNA expression in plasma [61]. Instead, Kobayashi *et al* [63] found out a large number of exosomes with high content of let-7 miRNAs, in particular, let-7a, b, c, d, e, f in the *in vitro* study.

No correlation has been found between let-7f plasma expression and overall survival, however low miRNA level correlated with reduced recurrence-free survival [61]. Decreased expression of let-7b, d, i, c, e, f correlates with the poor prognosis [40]. Low let-7e expression, high Rad51 expression and BRAC1 are associated with unlikely recurrence and overall survival [53].

Zheng *et al* [61] compared the miRNA expression in plasma and tumour tissue of women with OC and healthy ovarian tissue controls. There was a difference with control in 30 plasma miRNAs, including hypoexpressed: let-7a, d, e, f, miR-98; and hyperexpressed: let-7f-2. Such data is confirmed in another study, where serum let-7b was detected in a hypoexpressed state in patients with OC [64].

Available data states that let-7 family has predominantly oncosuppressive properties – let-7 expression in peripheral blood was indicated as a positive prognostic factor for patients with OC (Table 7).

## Conclusions

Despite the controversial results of experiments on the role of miRNA families 200 and let-7 in OC, their potential benefit as markers of early OC diagnosis and its prevalence remains encouraging. The miRNA-200 family is presented as a regulator of EMT and its counterpart – MET through impacting ZEB1/ZEB2 expression. Disruption in this process can lead to failure in cell plasticity and promote cancer metastasis. Thus, early detection of miRNA-200 family dysregulation in blood through ‘liquid biopsy’ could be beneficial for the timely OC diagnostic. miR-200 family regulation impact on EMT-MET mechanism in the scope of OC treatment remains to be addressed by further research.

miRNA family of let-7 in the scope of OC is studied insufficiently. Available data states that it has predominantly oncosuppressive properties. Decreased expression of let-7e, f, d, c, a-e, i, a, b in tumour samples and decreased let-7f, d, a-e in cell lines, decreased cell growth and reduced lymph node involvement prove an oncosuppressive role of this miRNAs family. All available data support the oncosuppressive role of the family – let-7 expression in peripheral blood was indicated as positive prognostic factor for patients with OC.

For both miR-200 and let-7 family, data concerning their role in carcinogenesis of OC appeared to be different during the evaluation of blood and tumour tissue expression. F.i. miR-200a and let-7a expression in tumour tissue represent them mostly as carcinogenic miRNAs. However, their expression in serum samples proved their oncosuppressive abilities. miR-200 and let-7 family role discrepancies are caused by various differences in research planning. Research groups used different methods to store biomaterial. This is crucial. F.i. fixation in formalin may hinder miRNA level in tumour tissue. Similarly, measurement of only exosome fraction may not represent the whole extracellular miRNA scope. Furthermore, no absolute ranges for miR-200 and let-7 family expression have been established yet, which hinder the standard evaluation of miRNA expression and its implementation into clinical practice.

The miRNAs of the 200 and let-7 families are promising for their further use in clinical practice, but the mechanisms of their involvement in cancer therapeutic response require additional research. The procedure of miRNA extraction and evaluation should be standardised in order to provide reliable data for clinical use. Future studies in the field, once incorporated these suggestions, may eliminate or explain the existing contradictions concerning the role of these miRNAs in the process of carcinogenesis, prognosis and impact on chemosensitivity in patients with OC. This will complement the current knowledge of OC molecular and cell biology, have an immense impact on the development of accurate, time- and cost-efficient therapeutic progression assessment and motivate new strategies for OC clinical treatment in the future.

## List of abbreviations

OC, Ovarian cancer; miRNA, miR, microRNA; FFPE, Formalin-fixed paraffin-embedded; MAPK14, Mitogen-activated protein kinase 14; CA-125, Cancer antigen 125; TUBB3, Tubulin beta chain 3 gene; Lin28B, Lin-28 homolog B; IGF-II, Insulin-like growth factor 2; MDR1, Multidrug resistance mutation 1; EMT, Epithelial-mesenchymal transition; MET, Mesenchymal-epithelial transition; ZEB1, Zinc finger E-box-binding homeobox 1; ZEB2, Zinc finger E-box-binding homeobox 2; f.i., For instance.

## Conflicts of interest

The authors declare that they have no conflicts of interest.

## Funding statement

The authors received no financial support for the research or authorship of this paper. This publication was supported by *e*cancer Global Foundation.

## Figures and Tables

**Table 1.** Data supporting the oncosuppressive role of miR-200a.

Deregulation	Clinical value	Research material	References
↑	Prolonged survival. Expression decreases along with the disease progression.	Tissue samples	Eitan *et al* [25]
↑	↑ sensitivity to taxanes.	*In vitro*, *In vivo*	Liu *et al* [29]
↑	Prolonged overall and recurrence-free survival. ↑ sensitivity to taxanes.	*In vitro*, *In vivo*	Mateescu *et al* [27]
↓	Reduced overall survival. Expression is higher in stages I–II, decreases along with the metastatic spread to lymph nodes, occurrence of ascites. There is no correlation between grade, size, histological subtype of tumour and the miRNA expression.	*In vitro*, tissue samples	Sun *et al* [20]
↓	OC expression > benign changes. Stages I–II > Stages III–IV. Intact lymph nodes > metastatically affected lymph nodes. Low-grade > high-grade. There is no correlation with the histological subtype.	*In vitro*, tissue samples	Xu *et al* [13]
↓	Reduced overall and recurrence-free survival.	*In vitro*, tissue samples	Hu *et al* [11]

**Table 2.** Data supporting the carcinogenic role of miR-200a.

Deregulation	Clinical value	Research material	References
↑	Reduced overall and recurrence-free survival.	Tissue samples	Nam *et al* [31]
↑	High-grade OC. Stages III–IV.	*In vitro*, tissue samples	Yang *et al* [17]
↑	OC expression > healthy patients. Stages III–IV > Stages I–II. Metastatically affected lymph nodes > Intact lymph nodes. High-grade > low-grade.	*In vitro*, tissue samples	Zhu *et al* [18]
↑	Expression increases in OC, metastatic spread to lymph nodes. There is no dependence on a grade.	*In vitro*, tissue samples	Suo *et al* [21]
↑	Expression is higher in stages III–IV, high-grade tumours. There is no correlation with the histological subtype, residual tumour masses or recurrence.	*In vitro*, *in vivo*, tissue samples	Cao *et al* [19]
↑	OC expression > healthy cells.	*In vitro*	Wyman *et al* [24]

**Table 3.** Data supporting the oncosuppressive role of miR-200c.

Deregulation	Clinical value	Research material	References
↑	Decreased recurrence rate and progression.	Tissue samples	Leskelä *et al* [28]
↑	Increased sensitivity to paclitaxel. Enhanced proliferation, inhibition of migration and cell invasion.	*In vitro*	Cochrane *et al* [14]
↑	Depending on the location of the HuR protein in the cell, it causes different outcomes: when located in the nucleus of a cell, it causes an increase in sensitivity to paclitaxel and cisplatin, and, consequently, a better prognosis for patients; cytoplasmic location predetermines an unfavourable prognosis.	In vitro	Prislei et al [16]
↓	Decreased sensitivity to cisplatin.	*In vitro*, *in vivo*, tissue samples	Liu *et al* [33]
↑	Increased sensitivity to paclitaxel, decrease of carboplatin.	*In vitro*	Brozovic *et al* [45]
↑	Hypersensitivity to paclitaxel, epothilone B, vincristine. No effect on cisplatin, doxorubicin, mitomycin C. Reduces migration and invasion. Does not affect proliferation	*In vitro*	Cochrane *et al* [47]
↑	Increased sensitivity to paclitaxel.	*In vitro*	Cittelly *et al* [15]
↑	Expression is higher in stages I–II of OC, with intact lymph nodes. Hyperexpression suppresses migration and invasion.	*In vitro*, *in vivo*, tissue samples	Lu *et al* [23]
↓	Decreased overall and recurrence-free survival.	Tissue samples	Marchini *et al* [32]

**Table 4.** Data supporting the carcinogenic role of miR-200c.

Deregulation	Clinical value	Research material	References
↑	Decreased overall and recurrence-free survival.	Tissue samples	Nam *et al* [31]
↑	Decreased overall and recurrence-free survival. There is no correlation with the level of CA-125.	Tissue samples	Elgaaen *et al* [34]
↑	Decreased overall survival. Higher expression in stages III–IV. No correlations were found with the grade, histological subtype of OC, tumour size and age of patients.	Tissue samples	Cao *et al* [19]

**Table 5.** The clinical value of mir-200b, -141, -429.

miRNA	Clinical value	Reference
miR-200b	- causes reduced overall and recurrence-free survival.	Nam *et al* [31]
	- reduces overall survival; expression is increased in stages III–IV, high-grade OC. - there is no correlation with the age of patients, size and histological subtype of the tumour.	Cao *et al* [19]
	- probably increases sensitivity to paclitaxel.	Leskelä *et al* [28]
	- increased expression increases sensitivity to cisplatin.	Liu *et al* [33]
	- expression in OC is higher than in healthy cells.	Wyman *et al* [24]
	- expression is higher in stage I, as compared to III.	Eitan *et al* [25]
miR-141, -429 miR-141 miR-429	- ↑ expression reduces the overall and relapse-free survival.	Nam *et al* [31]
	- expression in cancer cells is higher than in healthy ones.	Wyman *et al* [24]
	- ↑ expression prolongs overall and relapse-free survival and increases sensitivity to paclitaxel.	Mateescu *et al* [27]
	- ↑ expression increases sensitivity to paclitaxel and decreases it to carboplatin.	Brozovic *et al* [45]
	- causes resistance to platinum drugs.	Van Jaarsveld *et al* [46]
	- ↓ expression reduces overall and relapse-free survival.	Leskelä *et al* [28]
	- ↑ expression increases sensitivity to cisplatin.	Wang *et al* [48]

**Table 6.** Clinical value of miRNAs of the let-7 family.

miRNA	Clinical value	Reference
Let-7i	- reduced expression shortens recurrence-free survival. - reduces sensitivity to cisplatin.	Yang *et al* [37]
	- increased expression increases sensitivity to paclitaxel.	Liu *et al* [49]
	- reduced expression causes a worse prognosis for the patient.	Helland *et al* [40]
Let-7a	- hyperexpression reduces overall survival, sensitivity to platinum and taxanes.	Lu *et al* [43]
	- hyperexpression increases the sensitivity to platinum drugs. - reduces the sensitivity to taxanes.	Lu *et al* [42]
	- reduces sensitivity to taxanes, doxorubicin, interferon α.	Tsang and Kwok [51]
	- let-7a should be considered together with Lin28B and IGF-II, of which Lin28B is the main element with the worst prognostic value in hyperexpression.	Lu *et al* [44]
Let-7d,c	- reduced expression causes a worse prognosis for the patient.	Helland et al [40]
Let-7e	- reduced expression shortens recurrence-free and overall survival. - reduces sensitivity to cisplatin.	Xiao *et al* [53]
	- increases sensitivity to cisplatin.	Kuang *et al* [54]
	- increased expression reduces sensitivity to paclitaxel.	Sorrentino *et al* [52]
	- reduced expression causes a worse prognosis for the patient.	Helland *et al* [40]
Let-7d	- reduced expression causes a worse prognosis for the patient.	Helland *et al* [40]
	- proves to be an oncosuppressive miRNA.	Boyerinas *et al* [55]
Let-7g	- prolongs recurrence-free survival. - increases sensitivity to paclitaxel and vinblastine, does not affect sensitivity to carboplatin.	Boyerinas *et al* [55]
Let-7f	- reduced expression causes a worse prognosis for the patient.	Helland *et al* [40]

**Table 7.** Expression of miRNAs in the blood and their clinical value.

miRNA	Clinical value	Reference
miR-200a	- increased serum expression does not correlate with overall or relapse-free survival, CA-125 levels, tumour size and neoadjuvant treatment.	Kan *et al* [30]
	- expression in OC > benign tumours. - expression does not depend on the stage of OC. - there is no correlation with the OC grade.	Meng *et al* [56]
	- expression does not depend on the stage of OC. - expression in healthy women > benign ovarian tumours. - there is no correlation with the involvement of lymph nodes in the process.	Meng *et al* [35]
	- expression increases depending on the stage of OC and metastatic spread. - no correlation with the lymph node involvement in the process, grade, CA-125 level or tumour size was observed.	Zuberi *et al* [57]
miR-200c	- increased expression does not correlate with the overall or relapse-free survival, CA-125 levels, tumour size, CA-125 levels, tumour size and neoadjuvant treatment.	Kan et al [30]
	- increased expression correlates with reduced overall and relapse-free survival, lymph node involvement and CA-125 levels. Increased expression was observed for stages III–IV.	Meng et al [35]
	- expression of stages I–II < stages III–IV and correlates with the involvement of lymph nodes.	Meng *et al* [56]
	- expression of stage I <stage II <stages III–IV and correlates with lymph node involvement, metastatic spread.	Zuberi *et al* [57]
	- expression of stages I–II > stages III–IV and increases with metastatic spread. - does not correlate with lymph node involvement, CA-125 level and serum expression does not coincide with tissue expression.	Wang *et al* [60]
	- increased expression prolongs overall survival. - expression of stages I–II > stages III–IV, decreases with the metastatic spread.	Gao and Wu [26]
miR-200b	- shortens the overall survival. - correlates with the lymph node involvement and CA-125 level. - expression is increased in stages III–IV.	Meng *et al* [35]
	- expression is higher in OC compared to healthy controls.	Meng *et al* [56]
	- expression of healthy < benign tumours < OC and correlates with CA-125. - there is no correlation with the progression of OC or grade.	Kan *et al* [30]
	- no correlation with the tumour grade, CA-125, OC progression, age, concentration of Hb in blood, absence of pregnancies and menopausal status.	Zuberi *et al* [57]
miR-141	- expression increases with the disease progression in cases of metastatic spread and adversely affects overall survival.	Gao and Wu [26]
	- exosomes were not detected in serum.	Meng *et al* [35]
	- expression in serum is reduced.	Zheng *et al* [61]
miR-429	- expression increases with the disease progression. - correlates with CA-125. - inversely affects the overall survival. - exosomes were not detected in serum.	Meng *et al* [62]
		Meng *et al* [35]
Let-7i	- expression in serum is reduced.	Langhe *et al* [41]
Let-7a	- hypoexpressed in plasma.	Zheng *et al* [61]
	- increased amount in exosomes.	Kobayashi *et al* [63]
Let-7d,e,f	- hypoexpressed in the blood serum. - for let-7f, hypoexpression correlates with the reduced recurrence-free survival; there is no dependence on overall survival. - let-7f-2 is in a hyperexpressed state.	Zheng *et al* [61]
Let-7b	- hypoexpressed in the blood.	Chung *et al* [64]
